# Understanding pup affective state through ethologically significant ultrasonic vocalization frequency

**DOI:** 10.1038/s41598-017-13518-6

**Published:** 2017-10-18

**Authors:** Julie Boulanger-Bertolus, Millie Rincón-Cortés, Regina M. Sullivan, Anne-Marie Mouly

**Affiliations:** 1 Lyon Neuroscience Research Center, INSERM U1028; CNRS UMR5292; University Lyon1, Lyon, France; 2 Emotional Brain Institute, Nathan Kline Institute, Child and Adolescent Psychiatry, New York University School of Medicine, New York, NY USA; 3 Present Address: University of Michigan, Ann Arbor, USA

## Abstract

Throughout life, rats emit ultrasonic vocalizations (USV) when confronted with an aversive situation. However, the conditions classically used to elicit USV vary greatly with the animal’s age (isolation from the dam in infancy, versus nociceptive stimulation in adults). The present study is the first to characterize USV responses to the same aversive event throughout development. Specifically, infant, juvenile and adult rats were presented with mild foot-shocks and their USV frequency, duration, and relationship with respiration and behavior were compared. In juvenile and adult rats, a single class of USV is observed with an age-dependent main frequency and duration (30 kHz/400 ms in juveniles, 22 kHz/900 ms in adults). In contrast, infant rat USV were split into two classes with specific relationships with respiration and behavior: 40 kHz/300 ms and 66 kHz/21 ms. Next, we questioned if these infant USV were also emitted in a more naturalistic context by exposing pups to interactions with the mother treating them roughly. This treatment enhanced 40-kHz USV while leaving 66-kHz USV unchanged suggesting that the use of USV goes far beyond a signal studied in terms of amount of emission, and can inform us about some aspects of the infant’s affective state.

## Introduction

Rats and other rodents have developed communication in the ultrasonic range of sound frequencies. Adult rats emit two types of ultrasonic vocalizations (USV) that can be discriminated by their frequencies and durations. Long “22-kHz” vocalizations (18–32 kHz) are observed in aversive situations such as male-male aggression and social defeat^1,2^, exposure to predator^3^ or inescapable painful stimuli^4^. They are thought to reflect a negative affective state of the animal^5^. In contrast, rats emit short “50-kHz” vocalizations (35–72 kHz) in positive situations as induced by social play^5,6^, during sexual interactions^7^ or during gentle tactile stimulation^8^. 50-kHz vocalizations are thus considered as an index of a positive affect^5^.

Ultrasonic vocalizations are also emitted by rat pups from the day after birth to the time of weaning^1,9,10^ (reviewed in^11^). Classically described infant USV occur in the 40-70-kHz range, and are commonly referred to as “40-kHz” USV. They have been thoroughly investigated within the context of isolation from the mother and nest. Indeed, separation of a single pup from its littermates and dam in a novel test chamber (isolation) elicits high rates of USV in rat pups. Isolation is by far the most frequently used and potent paradigm to induce USV in experimental studies and infant isolation USV have been shown to elicit maternal retrieval responses^12^ suggesting that rat pups USV may function as calling signals^13,14^.

Because the peak energy frequency of USV emitted by pups is approximately 40 kHz, many authors have used bat detectors tuned to 40–50 kHz or bandpass filters centered around these frequencies^9,15–25^. However using the broadband output signal, Brudzynski *et al*.^26^ reported that 10- to 17-day-old rat pups were able to emit vocalizations with peak frequency ranging from 5 kHz to 130 kHz. Thus, pups are emitting USV in broad ranges, suggesting a greater complexity of call signals than previously considered.

The approach to understanding USV production in infancy and adulthood has focused on age relevant stimuli presentations within an ethologically relevant context. While this approach is valuable and has provided enormous understanding of ethologically relevant contexts for USV production, it also hinders developmental comparison of the USV. Here we take a two-pronged approach to further our understanding of USV. First, we used the same aversive stimulus (foot-shock) across development and compared USV in response to this stimulus at three different ages: infant, juvenile and adult. This allowed us to describe the existence of two classes of USV in infant rats: one corresponding to the infant USV classically described in the literature and showing similarities with adult and juvenile USV, and a novel class, poorly characterized so far, which appears to be specific to infancy. Second, we used a naturalistic and relevant adverse context in infant rats by exposing pups to a mother showing rough maternal behaviors used as a model of maltreatment of infants, with limited nursing^27–30^. We assessed whether the two classes of USV observed in the context of foot-shock delivery were also induced by this naturalistic aversive context.

## Results

### USV in response to the same aversive event vary greatly throughout ontogeny

**Figure 1:**
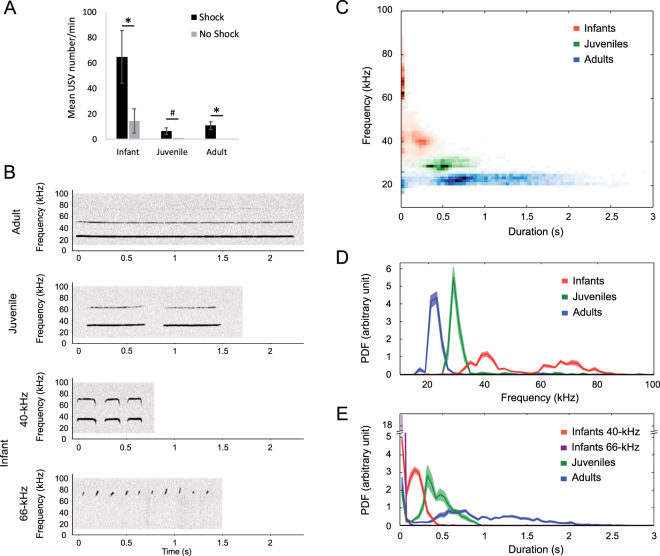
USV as a function of age. (**A**) At all ages, animals emit significantly more USV when shocks were delivered during the session than in the absence of shocks (Two factors ANOVA, Shock [F(1,43) = 9.88, p = 0.003], age [F(2,43) = 10.99, p < 0.001], Shock x Age interaction |F(2,43) = 4.07, p = 0.024]; Post-hoc comparison Shock vs NoShock: Infants t(14) = 2.39, p = 0.032; Juveniles t(13) = 1.92, p = 0.077; Adults t(16) = 4.00, p = 0.001; Infant vs juvenile: t(29) = 2.64, p = 0.013, vs adult: t(32) = 2.75, p = 0.010, Adult vs Juvenile: t < 1). *p < 0.05, ^#^p < 0.10. (**B**) Examples of individual sonograms of USV at all ages. (**C**) The frequency and duration of each USV are measured in infants, juveniles and adults and a 2-dimensional Probability of Density Function (PDF) is calculated over all the USV of the age group. The intensity of the color represents the relative amount of USV with the corresponding frequency and duration. (**D**) PDF ( ± SEM) of USV frequency: in adult animals, USV frequency peaked at 22.6 ± 0.3 kHz (mean ± SEM), in juvenile rats, at 29.7 ± 0.5 kHz and in infants, the lower frequency USV peaked at 40.5 ± 0.6 kHz while the higher frequency USV peaked at 66.4 ± 1.2 kHz. (**E**) PDF ( ± SEM) of USV duration: in adult animals, USV lasted 900 ± 100 ms, in juvenile rats, 450 ± 40 ms and in infants, the lower frequency USV lasted 140 ± 10 ms, while the higher frequency USV had very short durations (21 ± 2 ms).

To compare USV emission throughout ontogeny, we exposed infant from postnatal (PN) day 12–15, juvenile (PN22–24) and adult (over PN75) rats to the same aversive stimulus (0.4-mA, 1-s foot-shock). Rats were isolated in an experimental cage kept at age-dependent thermo-neutral temperature and submitted to 5 foot-shocks throughout a 30-min session. Control animals were isolated in the experimental cage but did not receive any shock. The data showed that foot-shock delivery enhanced the amount of USV emitted at all ages compared to control unshocked animals (see Figure 1A).

USV varied greatly in frequency and duration during ontogeny in response to the same foot-shock parameters. Specifically, visual inspection first revealed that in adult and juvenile rats, a single type of USV is observed, while there were two classes of USV observed in infants (see examples on Fig. 1B). The representation of the USV frequency function of the duration detected age-specific differences across development, with juvenile and adult USV occurring as a single cluster on this distribution, while infant USV were shorter and split into two clusters (Fig. 1C). Further analysis of frequency and duration revealed that in adult animals, USV frequency peaked between 20 and 25 kHz (mean ± SEM: 22.6 ± 0.3 kHz) (Fig. 1D) and their duration was widely spread between 400 and 2000 ms (900 ± 100 ms) (Fig. 1E). In juvenile rats, the USV occurred at a slightly higher frequency, peaking at about 30 kHz (29.7 ± 0.5 kHz) and were of a shorter duration, between 250 to 750 ms (450 ± 40 ms). In infants, the lower frequency USV peaked between 35 and 45 kHz (40.5 ± 0.6 kHz) and lasted for about 200 ms (140 ± 10 ms), while the higher frequency USV peaked between 60 and 80 kHz (66.4 ± 1.2 kHz) and had very short durations (21 ± 2 ms). These two kinds of infant USV can either co-occur or be emitted separately (see Figure S1) and will be referred to as “40-kHz” and “66-kHz” USV respectively.

### 40-kHz and 66-kHz Infant USV exhibit different relationships with respiration

In adult rats 22-kHz USV are typically observed when the rat is freezing. In addition, they are emitted almost exclusively during expiration and affect its length and shape^31–33^. Here, we examined the relationship between USV calls, behavior and respiration at the different ages.

**Figure 2:**
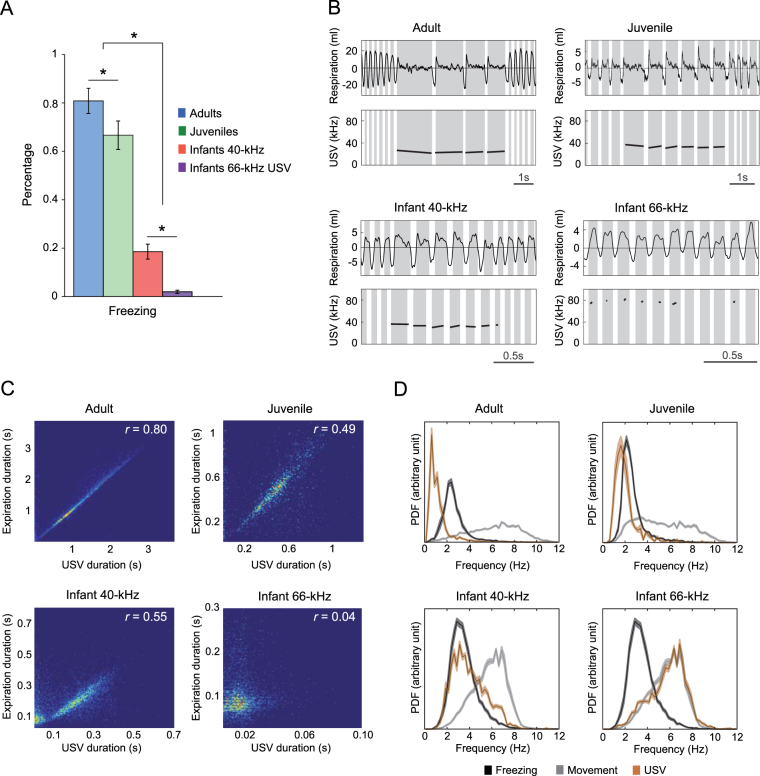
Link between respiration, behavior and USV. (**A**) Percentage of USV emitted when the rat is freezing versus moving at the different ages of development. Adult and juvenile rats emit USV mainly during freezing while infants mostly move when vocalizing [ANOVA, Age effect: F(3,22) = 121.10, p < 0.001]. *p < 0.05. (**B**) Individual examples of typical respiratory signals (top panels) observed during ultrasonic vocalizations (bottom panels). Inspiratory and expiratory phases are depicted in white and grey respectively. For adults, juveniles and infants 40-kHz USV, the USV lasts as long as the expiratory phase of the respiratory cycle. The infants’ 66-kHz USV calls, however, are shorter than the expiratory phase. (**C**) Correlation between USV duration and expiration duration at the different ages. For adult, juvenile and infant 40-kHz USV, USV duration is correlated with expiration duration (Adult: r = 0.80, n = 3945; Juvenile: r = 0.49, n = 1927; Infant 40-kHz USV: r = 0.55, n = 5479), while this is not the case for infant 66-kHz USV (r = 0.04, n = 3270). (**D**) PDF (i.e. distribution; ± SEM) of the respiratory frequency during silent freezing (black curve) and movement (grey curve), and during USV emission (colored curve). The respiratory frequency is decreased during USV emission for adult, juvenile and 40-kHz infantile USV compared to that observed during the associated behavior (freezing for adults and juveniles and moving for infants). In contrast, the respiratory frequency of infant rats is not affected by the emission of 66-kHz USV.

We first assessed which behaviors occurred during USV emission at the three ages. In the context of shock delivery, the animals’ behavioral repertoire was mainly limited to either freezing or moving. Therefore, we quantified the percentage of USV emitted during movement vs. freezing for each developmental age. In adults and juveniles, most of the USV were emitted during freezing (Fig. 2A). In contrast, infant USV were preferentially (for 40-kHz USV) or almost exclusively (for 66-kHz USV) emitted during movement.

We then assessed the impact of USV emission on the animals’ respiratory rate. Our data in adults confirmed previous results^31–33^ showing that the emission of USV strongly alters the respiratory signal primarily through a lengthening of expiration (Fig. 2B). Here we show that it is also the case for juvenile 30-kHz and infant 40-kHz USV emission. In contrast, the emission of 66-kHz infant USV does not alter the respiratory signal. Further comparison of the USV and expiration durations at the different ages highlighted that, while infant 40-kHz USV lasted for the whole expiratory cycle duration, as seen in adults and juveniles, 66-kHz USV were shorter in duration than expiration (correlation shown in Fig. 2C). As a result, in adult and juvenile rats, for which USV are essentially observed during freezing, the emission of USV induces a lowering of respiratory rate below that observed during silent freezing (Fig. 2D). In infants, for which USV are essentially observed during movement, the emission of 40-kHz USV induces a lowering of respiratory rate below that observed during movement while the emission of 66-kHz did not alter the ongoing respiratory rate.

In summary, USV in response to the same aversive event (foot-shock delivery) exhibit clear-cut differences between infants, juveniles and adults (summary in Supplementary Table 1). Importantly, this experiment revealed that infant USV can be divided in two categories: 40-kHz USV which present similarities with juvenile and adult USV, and 66-kHz USV which seem more distinct. Because infant 66-kHz USV in response to mild foot-shocks had not been described in the literature so far, we assessed whether they are also observed in a more naturalistic aversive context.

### 40-kHz and 66-kHz Infant USV are differentially modulated by rough maternal interactions

**Figure 3:**
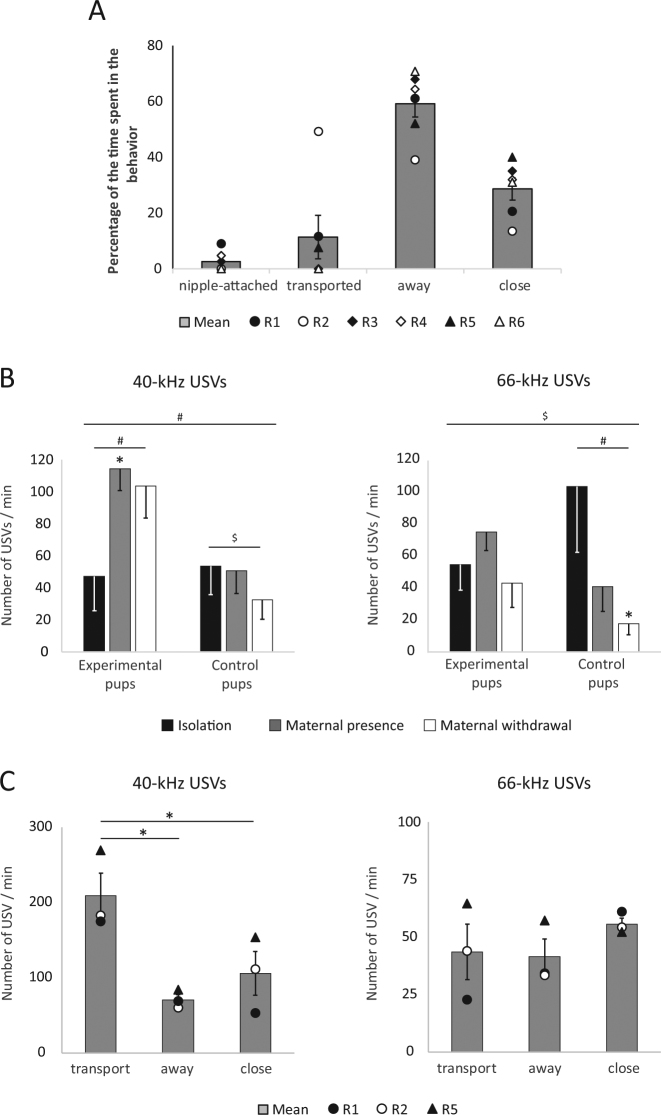
USV emitted in naturalistic aversive situation. (**A**) When the dam is introduced in an unfamiliar cage containing the pup, it spends more time away from the pup and shows minimal nursing behavior. When nursing occurred, it was brief and typically ended with the dam walking away dragging the pup attached to the nipple. In addition, the dam was observed in atypical transport of the pup (up to 50% of the time for pup R2), a behavior that can be painful for the pup (individual points and mean ± SEM). (**B**) Interaction with the dam (Maternal presence) enhanced 40-kHz USV rate compared to isolation levels [two factors ANOVA; independent factor Group: F(1,10) = 5.10, p = 0.048; repeated factor Period: F(3,20) = 3.20, p = 0.062; Group x Period: F(2,20) = 5.62; p = 0.012; Experimental group, n = 6: F(2,10) = 4.48, p = 0.041; Control group, n = 6: F(2,10) = 3.82, p = 0.059] and prevented the decrease in 66-kHz USV rate observed in the control group throughout the session [Group: F < 1; Period: F(2,20) = 3.10, p = 0.067; Group x Period: F(2,20) = 2.71, p = 0.091; Experimental group: F < 1; Control group: F(2,10) = 5.62, p = 0.023]. ^#^p < 0.05 for the ANOVA; ^$^p < 0.1 for the ANOVA. *p < 0.05 post-hoc analysis compared to Isolation period. (**C**) Transportation of the pups, when occurring, enhanced the 40-kHz USV rate compared to when the dam was not interacting with the pup [F(2,4) = 13.34, p = 0.017], but it did not affect the amount of 66-kHz USV emitted [F(2,4) = 1.29, p = 0.369]. *p < 0.05 post-hoc analysis.

**Figure 4:**
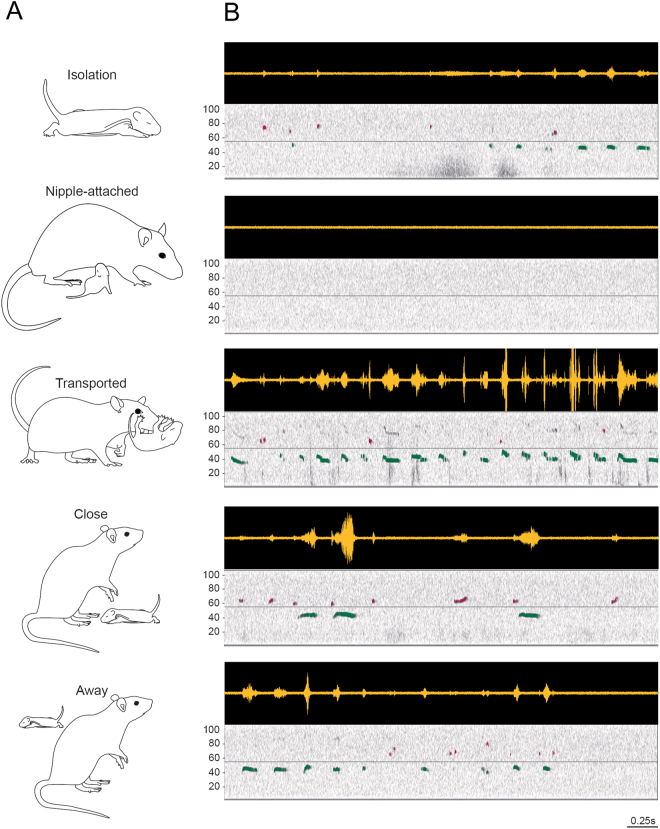
Examples of infant USV emitted in naturalistic situations. (**A**) The different behavioral states of the pup. B. Examples of USV recorded during each behavioral state. Top panels represent the raw USV signals and bottom panels the associated spectrograms (frequency in kHz as a function of time). The light horizontal grey line on the spectrogram represents the separation between 40-kHz (green) and 66-kHz USV (purple). Rough transport of the pup by the dam (transport by a limb or the belly skin) enhances the emission of 40-kHz USV.

The dam is the most prominent stimulus that an infant rat encounters in its first days of life and the quality of maternal care likely modifies the emotional state of the infant and affects pups’ USV call rate^11^. To induce naturalistic noxious stimulation of pups, we used an experimental procedure previously documented to produce maternal rough handling of pups. Rat pups were exposed to their mother one at a time in an unfamiliar bedding-free plexiglass cage. In this context, the mother spent most of her time exploring the cage, occasionally stepping on the pup, rarely nursing it, and frequently roughly transporting it^27,28^ (Fig. 3A). USV emitted by the pup in these experimental conditions were quantified during three 5-min periods corresponding to isolation from the dam (Isolation), introduction of the active dam (Maternal presence) and removal of the dam (Maternal withdrawal), and compared to USV recorded in control animals for which no dam was introduced. The data showed that in the two groups, both 40- and 66-kHz USV are emitted by infant rats upon isolation from the dam and the nest. Introduction of the dam in the cage (only in experimental pups) significantly enhanced infant 40-kHz USV rate which remained high after maternal withdrawal, while in control pups 40-kHz USV rate tended to decline throughout the session (Fig. 3B). In contrast, introduction of the dam did not enhance 66-kHz USV rate although it prevented the decrease observed in the control group (Fig. 3B). Further analysis was done on pup vocalizations during maternal transport of pups (*n* = 3). Our naturalistic manipulations produced atypical maternal transport behavior and pups were carried either by the limbs or the belly skin, rather than the typical nape of the neck. This replicates models of abusive maternal caregiving which, if repeated, produces atypical neurobehavioral development and pathology^27–30^. Our analysis suggests that this atypical maternal transport was the most robust parameter to enhance 40-kHz USV emission compared to pups not being transported. This rough transport did not affect 66-kHz USV emission (Fig. 3C, examples on Fig. 4). While this effect is statistically significant [*F*(2,4) = 13.34, *p* = 0.017], due to the small number of transported pups, this will need to be confirmed in future experiments.

## Discussion

When placed in an aversive situation, rats emit USV. While adult rats emit long 22-kHz in situations such as male-male aggression and social defeat^1,2^, exposure to predator^3^ or inescapable painful stimuli^4^, rat pups emit vocalizations in the broadly defined 40-70-kHz range when isolated from the mother and nest^1,9–11^. The present study is the first to provide an in-depth characterization of USV in response to the same aversive event (foot-shock delivery) throughout development. The data show that in juvenile and adult rats, a single class of USV is observed in response to shock as characterized by an age-dependent main frequency and duration (30 kHz/400 ms in juveniles and 22 kHz/900 ms in adults). In contrast, infant rats’ USV were split in two classes characterized by distinct frequency and duration: 40 kHz/140 ms and 66 kHz/21 ms respectively. The two types of infant USV were also observed in a more naturalistic, aversive situation as assessed by exposure to rough maternal interactions. Importantly, in both experimental and naturalistic aversive contexts, the two types of USV present consistent differences, although the assessment of USVs in the presence of rough interaction with the mother highlights the diversity of USV expression within a social context. The novelty of the present study is thus to highlight the existence of heterogeneity within the classically broadly defined 40-70-kHz infant USV category, with the identification of two distinct types: 40-kHz versus 66-kHz USV with specific properties and its modulation within the social context of the mother-infant dyad.

Using the same aversive event across development to induce USV allowed us to compare USV characteristics. We describe a progressive decrease in shock-induced USV frequency from 40-kHz in infancy to 30-kHz in juveniles to 22-kHz in adults. This could be linked to the developmental increase in the size and length of the larynx and the vocal tract. Indeed, Riede^34^ showed that the geometry of the glottis explains most of the frequency modulations observed in adults. Moreover, Blumberg *et al*.^35^ demonstrated that there is a highly significant linear relationship (*r* ^2^ = 0.9) between body weight and the frequency of the 40-kHz USV emitted by infant rats between the ages of PN2 and PN20. In adult rats, 22-kHz USV are classically reported in response to aversive events (predator encounter, agonistic interaction, noxious stimuli^1–4^) and are considered part of the animal’s defensive repertoire^36^. The present data suggest that juvenile 30-kHz and infant 40-kHz USV may serve the same function.

For 22-kHz USV, we showed that in juveniles and adults, USV are predominantly produced during freezing, which is consistent with the literature. Indeed, in adult rats, previous studies have shown that while immobility may occur without 22-kHz USV, such USV generally do not occur without immobility^37–39^. It is well documented that USVs at this frequency are vibrations caused when expired air is forced through constricted vocal folds. It is likely that the constricted vocal folds may be facilitated during freezing. Furthermore, Walker and Carrive^38^ suggested that USV emission also needs thorax immobilization, itself a component of the freezing posture whose role is to stabilize both the lower and upper parts of the trunk. However, some examples of 22-kHz USV during locomotion have been described in the literature^40^ and were also observed in this study. The present study confirms that the production of USV is likely optimal during freezing but can also occur during walking in both adults and juvenile rats. Interestingly, in adults, 50-kHz USV are typically produced during locomotion since they occur during social interactions such as rough-and-tumble play in juveniles^6,41^ and mating in adults^42,43^, and have also been described during feeding and moving^40,44^, as well as during agonistic interactions^2,45^.

In sharp contrast to juvenile and adult 22-kHz USV, most infant USV are preferentially observed during movement: 66-kHz USV were emitted almost exclusively during movement, while 40-kHz USV might be seen during immobility but to a lesser extent than during movement. This is in agreement with the literature. Indeed, Wöhr and Schwarting^46^ showed that infant ultrasonic calling was highly correlated with locomotor activity, especially in the case of pivoting behavior (360° rotations of the body). Haack *et al*.^47^ suggested that the circular pivoting locomotion of isolated rodent pups provides a means of projecting the ultrasonic “beam” over a wide directional range.

The production of rat ultrasonic calls is an active and dynamic process. Both ultrasounds and audible cries are normally produced at the onset of expiration^33^. Coordinated brainstem mechanisms are involved in the preparation of the respiratory system and larynx. Rats first make an inspiration, the larynx is stabilized, and then vocal folds are tightly opposed leaving only a small opening through which forced expiration produces a whistle-like call^34,48,49^. This forced expiratory effort involves sharp contraction of the abdominal musculature and of the spinal extensor muscles^11^. From this it ensues a clear dependence of USV production on breathing. Previous studies have indeed shown that in adult rats, there is a strong correlation between expiration duration and USV duration for 22-kHz USV^31,32,50^. Here we compared the relationship between USV calls and respiratory cycles at the different ages of development. We showed that 40-kHz infant USV, similarly to 30-kHz juvenile and 22-kHz adult USV, last more than 100 ms and match the duration of expiration, impacting respiratory rate. In contrast, the 66-kHz infant USV are much shorter than 100 ms and occur within small portions of the expiratory phase, with no impact on respiratory frequency. Such observation can be paralleled with a report by Sirotin *et al*.^51^ showing that adult rats’ 50-kHz USV production is largely restricted to periods of active sniffing and produces only a modest drop in sniff rate.

With respect to infant USV, the two types of infant USV are not solely induced in response to shock delivery and were also shown to occur when pups received rough treatment from the mother. Specifically, both 66-kHz and 40-kHz infant USV were observed during isolation from the dam, during controlled shock delivery, as well as during interactions with the mother when pups were handled roughly. These data confirm the abundant literature on isolation-induced USV in infant rats^10,20,22,26,46^, and suggest both USV types reflect the pup’s distress^18,20,46^. However, here we extend these results to indicate that these two types of USV also present differences. First, they have specific relationships with respiration, with 40-kHz emission inducing a lengthening of expiration and, as a consequence, a slowing of respiratory rate, while 66-kHz emission does not affect respiration. In that sense 40-kHz USV behave more like 30-kHz juvenile and 22-kHz adult USV^31–33^. Second, the two types of infant USV are differentially modulated by rough interactions with the mother. Indeed, while 66-kHz USV are unchanged, 40-kHz USV are enhanced by noxious interaction with the mother^27,28,52^. Preliminary observations carried out on a small number of pups suggest that rough handling transport, which is a source of painful stimulation for the pup^27,28,52^, is the most efficient stimulus for enhancing 40-kHz USV. The ability of the mother to modify pups’ neurobehavioral response to shock and pain-associated maternal behaviors has previously been documented, such as the cortex, paraventricular nucleus and amygdala as measured by autoradiography, Fos and local field potentials^53–57^.

In this study, gross visual inspection of the infant USV calls recorded in response to shocks suggested that their acoustic structure is most of the time poorly frequency-modulated, thus leading to two categories instead of the ten reported by Brudzynski *et al*.^26^ using manual categorization. This could be due to our focus on aversive stimuli used in the present studies, compared to Brudzynski *et al*. isolation context. Interestingly, a greater number of frequency modulated infant USV were detected during the interaction with the mother, as is illustrated in Fig. 4 in the Transported condition. However, the limited number of litters used for the naturalistic experiment precludes USV categorization.

It is possible that infant 66-kHz USV had been poorly characterized in the literature because pre-tuned bat detectors or microphones with band-pass filters were used, which limited the recording of 66-kHz USVs^9,15–25^. However, some studies have described USV in infant rats that resemble the 66-kHz USV described here^9,21,26,58,59^. For instance Kim and Bao^60^ described 40 and 60 kHz USV elicited by isolation in 15-day-old rat pups. Noirot^61^ previously described two types of infant rat USV mainly based on their duration: clicks (produced by very short pulses, transients or sweeps) and whistles (produced by pulses staying in tune at an almost constant frequency for more than 5 ms). Moreover, Brudzynski *et al*.^26^ reported that 10- to 17-day-old rat pups were capable of emitting sounds as high as 130 kHz. Harmon *et al*.^58^ were the first to specifically describe infant USV with average frequency and duration similar to the 66-kHz USV described here. Interestingly, Ise and Ohta^21^ assessed the impact of environmental stimuli or pharmacological treatment on the infant USV frequency distribution. They showed that the USV frequency distribution presented two distinct peaks at 30 kHz and 50 kHz, and that the high-frequency component was sensitive to stress induced by low ambient temperature, as well as activation of the CRF and GABAergic systems. Another study by Blazevic *et al*.^59^ also reported the existence of a bimodal distribution of the USV frequencies in infancy and showed that 40-kHz and 66-kHz USV can be differentially modulated by pharmacological interventions, monoamine oxidase inhibition in their case. Interestingly, although mouse and rat USV characteristics are very different, bimodal distribution of isolation USV has also been reported in mouse pups^62,63^ suggesting some similarities between these two species’ vocalizations at young ages.

Based on the present data and the observation of Blazevic *et al*.^59^, we suggest that infant 66-kHz USV represent a distinct class of infant USV and propose that they might be an infantile version of the adult 50-kHz USV. Indeed, infant 66-kHz and adult 50-kHz USV exhibit a similar relationship with the animal’s behavior (they are both emitted when the animal is moving^6,40,41,44,64^) and respiration (they have little or no impact on the respiratory rate^51^). Although more frequently associated with positive experience^5–8^, 50-kHz USV have also been described in mildly aversive situations^2,65^. Taylor *et al*.^65^ suggest that a specific sub-class of adult 50-kHz USV could be associated with a mild aversive state while 22-kHz USV would reflect a more pronounced negative emotional response. Here, we showed that exposing pups to rough maternal interactions enhances 40-kHz USV rate while leaving 66-kHz USV unchanged, which supports the hypothesis that, 50-kHz and 22-kHz USV in adults, and 66-kHz and 40-kHz USV in infants may reflect increasing levels of negative affective state.

Beside its undisputed interest for understanding intraspecies communication^12–14,66^, rodent USV investigation has proven to provide a powerful tool to assess affect, motivation, and social behavior in animal models of pathologies^67,68^. Analysis of the quality rather than quantity of USV in mice pups has increased over the past few years and brought useful information potentially  relevant to  neurodevelopmental disorders^69–73^. A similar analysis of infant rat USV may provide researchers with a new tool to understand communication deficits in animal models. Performing an in depth analysis of USV characteristics, we were able to confirm the existence of two qualitatively different types of USV in infant rats, suggesting that the use of USV goes far beyond a signal studied in terms of amount of emission, and the ratio of the USV frequencies can inform us on the affective state of the infant. The recording of these two types of infant USV can thus be used as a novel tool by those interested in communication deficits and affective alterations in animal models of neurodevelopmental disorders^74^. Moreover, when distressed or separated, human babies, like infant rodents, produce extensive vocalizations, including crying. Although any comparison between species needs to be conducted with extreme caution, and cries of human infants and rat pups are produced by very different physiological mechanisms, the translational assessment of infant vocalizations may contribute to a better understanding of the effects of variations in the perinatal environment on infant neurobehavioral development (for a review, see^75^).

## Material and Methods

### Animals

The subjects were 61 male and female Long Evans rats born and bred at the Lyon Neuroscience Research Center (originally from Janvier Labs, France). Some of the respiratory recordings have been used in a previous study^76^. For pups, only one female and one male pup per litter per treatment/test condition were used for all experiments. Day of birth was considered PN0. Three groups of ages were used: post-natal day 12 to 15 (PN12–15, Infants), PN22–24 (Juveniles) and older than PN75 (Adults). Infant and juvenile animals were maintained with their litters up to the end of the experiments while adults were housed by pairs. All animals were housed at 23 °C and maintained under a 12 h light-dark cycle (lights on from 6:00 am to 6:00 pm). Food and water were available *ad libitum* and an abundance of wood shavings was supplied for nest building. All animals were maintained at age-dependent thermo-neutral temperatures during experiments. All experiments were conducted in strict accordance with the European Community Council Directive of September 22, 2010 (2010/63/UE) and the French National Committee (87/848) for care and use of laboratory animals, and were carried out under the approval of the Direction of Veterinary Service (#69000692). Care was taken at all stages to minimize stress and discomfort to the animals.

### Foot-Shock experiment

Six experimental groups were defined: Infant Shock group (n = 7), Infant No-Shock group (n = 9), Juvenile Shock group (n = 7), Juvenile No-Shock group (n = 8), Adult Shock group (n = 8), Adult No Shock group (n = 10). Only the individuals emitting more than 50 USV in the shock groups were kept for further analyses of the USV characteristics (infants n = 7, juveniles n = 7 and adults n = 8).

### Experimental setup

The apparatus consisted of a whole body customized plethysmograph cage (diameter 20 cm, Emka Technologies, France) for recording respiratory signal (see^32^ for further description of the plethysmograph). The bottom was equipped with a shock floor connected to a programmable Coulbourn shocker (Bilaney Consultants GmbH, Düsseldorf, Germany). The USV emitted by the rats were recorded by a condenser ultra-sound microphone (Avisoft-Bioacoustics CM16/CMPA, Berlin, Germany) inserted in the tower on the top of the plethysmograph cage. The height of the plethysmograph was adapted to the age of the animal in order to optimize the signal-noise ratio, leading to a height varying from 16.5 cm for infants to 30 cm for the adults. The plethysmograph was placed in a sound-attenuating cage (L 70 cm, W 60 cm, H 70 cm). The behavior was monitored using four video cameras placed at each corner of the sound-attenuating cage (B/W CMOS PINHOLE camera, Velleman, Belgium).

### Training paradigm

Adult rats were handled and familiarized to the conditioning chamber for 20 min during the 4 days preceding the beginning of the experiment. Juveniles received only one day of handling and habituation while infants, for which conditioning to context is not yet developed^77^, were not handled in order to minimize distress from maternal separation.

The aversive event applied at all ages consisted of a 1-s mild (0.4 mA) foot-shock delivered through the grid floor. During the first 10 min of the conditioning session, animals were allowed an adaptation period of free exploration. This was followed by 5 foot-shocks delivered with a 4-min inter-shock interval. Vocalizations, respiration and behavior were continuously monitored throughout the session. The animals were returned to their home cage after the session.

### Data acquisition and pre-processing

The respiratory signal collected from the plethysmograph was amplified and sent to an acquisition card (MC-1608FS, Measurement Computing, USA; Sampling rate = 1000 Hz) for storage and offline analysis. Using whole body plethysmograph setup, natural breathing signal appears as a periodic phenomenon showing alternating negative (inspiration) and positive (expiration) deflections. The detection of respiratory cycles was achieved using an algorithm described in a previous study^78^. This algorithm performs two main operations: signal smoothing for noise reduction, and detection of crossing zero points in order to define accurately the inspiration and expiration phase starting points. Artifacts were eliminated by determining a cut-off value for signal duration (see^32^ for further description). Momentary respiratory frequency was determined as the inverse of the respiratory cycle (inspiration plus expiration) duration.

The video signal collected through the four video cameras was acquired with homemade acquisition software using the Matrox Imaging Library and a Matrox acquisition card (Morphis QxT 16VD/M4, Matrox video, UK). The animal’s freezing behavior was automatically detected using a LabView homemade software that had been validated by comparison to hand scoring by an experimenter blind to the rats’ condition. Automatically detected freezing episodes were then manually controlled to exclude quiet immobility. Scoring of the freezing at the different ages followed the methods defined by Takahashi^79^, taking into account the immaturity of infants’ musculoskeletal system. In adults and juveniles freezing was scored whenever the rat exhibited an immobile posture with the back arched and the ventrum above the floor (crouched posture^80^). In infants, the immaturity of the musculoskeletal system impedes such a tonic posture. Therefore, infants were scored as freezing when they adopted an immobile posture with the head in a stationary, non-tilted position. At all ages, movement was scored each time the rat was not immobile/freezing. No distinction was made between stationary movement and locomotion.

The ultrasound microphone was connected to a recording interface (UltraSoundGate 116Hb, Avisoft-Bioacoustics) with the following settings: sampling rate = 214285 Hz; format = 16 bit^39^. Recordings were transferred to Avisoft SASLab Pro (version 4.2, Avisoft-Bioacoustics, Berlin, Germany) and a fast Fourier transform (FFT) was conducted. Spectrograms were generated with an FFT-length of 512 points and a time window overlap of 87.5% (100% Frame, FlatTop window). These parameters produced a spectrogram at a frequency resolution of 419 Hz and a time resolution of 0.29 ms. The acoustic signal detection was provided by an automatic whistle tracking algorithm with a threshold of −20 dB, a minimum duration of 5 ms and a hold time (minimum silence duration to determine that a call has ended) of 5 ms, to allow detection of the smallest calls. Because these minimum duration and hold times are very permissive, the accuracy of detection was verified call by call by an experienced user. USV analyses were restricted to the rats emitting more than 50 USV throughout the session to allow minimum biases in the distribution analyses. In our study, rats emitted composite calls at all ages. These calls presented a spectrographic appearance of separate calls and were produced during the same expiratory phase^81^. In the present study, vocal outputs detected on the spectrogram that were within 30 ms of an adjacent call were categorized as composite calls^51^. Using this categorization, we found that the following proportions of USV calls could be categorized as composite calls: Infants 17 ± 1.3% (mean ± S.E.M.); Juveniles 4.8 ± 1.6%; Adults 4.5 ± 1.1%. Composite calls were counted as one call when the number of USVs were analyzed and were not considered when call duration was analyzed.

The pre-processed data (respiration, USV, behavior) were then entered in a database^82^ and synchronized via a TTL synchronization signal generated at the beginning of each experimental session. The different data stored in the database could be visualized simultaneously and were analyzed using scripts in Python.

### Data analysis

#### Shock induced USV

The number of USV recorded when the rats received shocks was expressed per minute and compared between groups (Foot-shock vs. No Foot-shock) and age (Infants, Juveniles, Adults) using a two-way ANOVA with independent factors, followed by *post-hoc* comparisons when allowed by the ANOVA results. Significance was set at *p* < 0.05.

#### USV Characteristics

For the 1-dimension Probability of Density Function (PDF) analyses, the frequency and duration of the vocalizations were analyzed by defining their PDF for each animal using a bin of 2 kHz for the frequency and 0.1 s for the duration. The PDFs were then averaged within conditions and the standard error determined for each point. For the 2-dimension PDF, the USV of all individuals of the same age were pooled for analysis.

#### USV versus Behavior

The behavioral data corresponding to each USV call were extracted and their category (Freezing or Movement) examined. This allowed us to determine in which behavioral state the USV occurred preferentially. Differences were tested using Student t-tests.

#### USV versus Respiration

The respiratory signal corresponding to each USV call was extracted and analyzed. Specifically, the duration of expiration phase during each call was examined and correlated to the duration of the call. The PDF of the respiratory rate was analyzed while the animal was silently freezing and moving, or vocalizing using the same analysis protocol used for USV characterization.

### Maternal Interaction experiment

#### Testing paradigm

On the testing day, pups were brought to the experimental room (warmed at 28–30 °C) in their home cage with their littermates, the dam being taken to another cage before transport to avoid any stress transmission to the pups. Testing started with the experimental pup being gently put into a clean Plexiglas empty cage (40 × 25 × 18 cm). The pup was left alone for 5 min (Isolation period) after which the mother was introduced for 5 min (Maternal presence period). Introducing the dam into an empty unfamiliar cage has been shown to induce maltreatment in rats^27,28^. The mother was then removed and the pup remained alone for an additional 5-min period (Maternal withdrawal period). In the control group, no dam was introduced during the Presence period, thus leading to 3 consecutive 5-min isolation periods. The USV emitted by the pups throughout the session were recorded by a condenser ultra-sound microphone (Frequency range: 10 kHz-200 kHz, Avisoft-Bioacoustics CM16/CMPA, Berlin, Germany) positioned 30 cm above the experimental cage.

### USV analysis

The USV were analyzed as described above. The USV emitted by the pups throughout the session were classified in two categories: 40-kHz (0–56 kHz) and 66-kHz (56–100 kHz). The number of USV recorded in both categories during each 5-min period was expressed per minute and compared between groups and periods using a two-way ANOVA with group as an independent factor and period as a repeated measure, followed by *post-hoc* comparisons when allowed by the ANOVA results. Significance was set at *p* < 0.05.

### Behavior analysis

The behavior of the dam-pup dyad was manually scored using BORIS^83^. “Close” was defined when the head of the pup was less than 2 cm away from any part of the body (except tail) of the mother, and “away” when the head of the pup was more than 2 cm away from the mother. “Nipple-attached” was defined when the pup was in a nursing position under the dam.

## Electronic supplementary material

Supplementary information
