# Supplementary material for: Understanding pup affective state through ethologically significant ultrasonic vocalization frequency
Source: Sci Rep. 2017 Oct 18;7:13483. doi: 10.1038/s41598-017-13518-6 (PMC5647438; doi:10.1038/s41598-017-13518-6)
Supplement: Supplementary file 1 — Supplementary information [file 41598_2017_13518_MOESM1_ESM.pdf]

Understanding pup affective state through ecologically significant ultrasonic vocalization frequency

Julie Boulanger-Bertolus, Millie Rincón-Cortés, [#]Regina M. Sullivan, [#]Anne-Marie Mouly

Supplementary Informations

Supplementary Table

***Table 1**: USV characteristics as a function of age*

	Adult	Juvenile	Infant	
**USV Mean frequency (kHz)**	22.6 ± 0.3	29.7 ± 0.5	40.5 ± 0.6	66.4 ± 1.2
**USV Duration (ms)**	900 ± 100	450 ± 40	140 ± 10	21 ± 2
**USV vs Expiration duration**	=	=	=	<
**Main behavior during USV**	Freezing	Freezing	Movement	Movement

Supplementary Figure

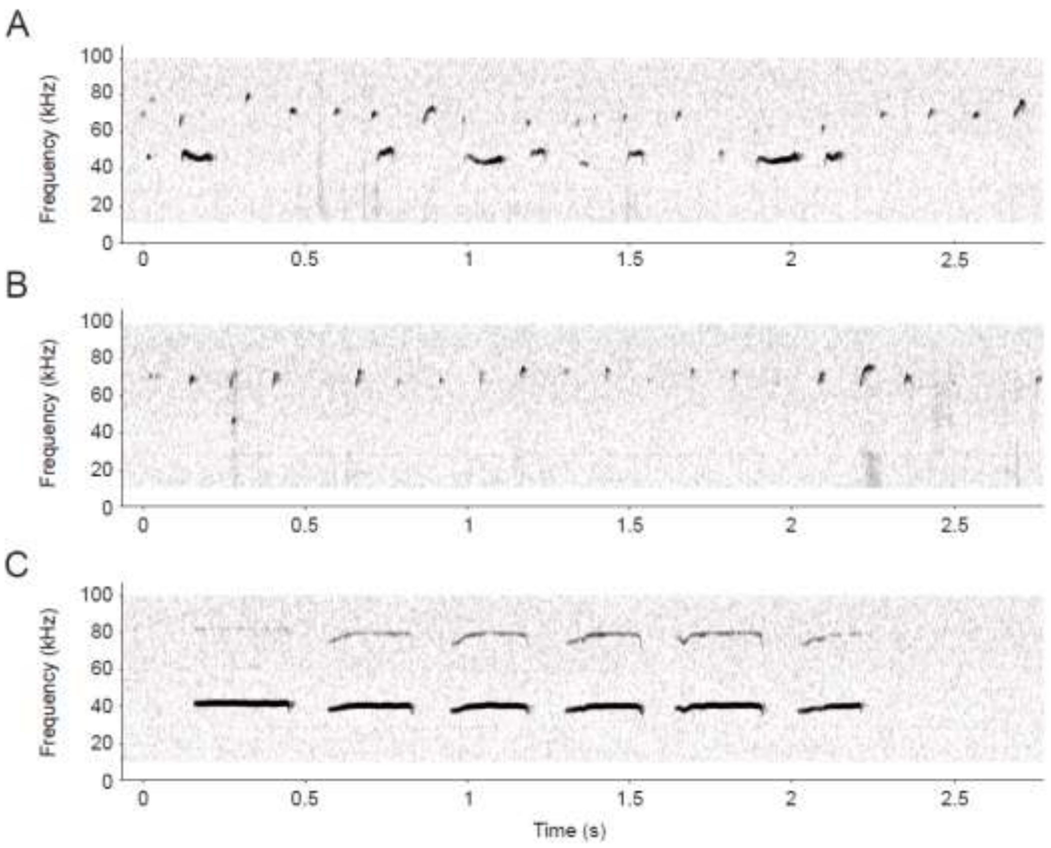

Supplementary figure 1: Two types of isolation USV are observed in infant rats. They can either co-occur (A) or be emitted by bouts of 66-kHz USV (B) and 40-kHz USV (C). Some harmonics may be visible on the spectrogram when 40-kHz USV are emitted.
